# Introduction of journal clubs for UG students of Ayurveda, a dire necessity

**DOI:** 10.1016/j.jaim.2024.100977

**Published:** 2024-10-14

**Authors:** Gaurav Soni

**Affiliations:** Department of Rachana Sharir, North Eastern Institute of Ayurveda & Homoeopathy (NEIAH), Mawdiangdiang, Shillong, East Khasi Hills, Meghalaya, 793018, India

Dear Sir,

Ayurveda formerly assumed as the traditional healing system of the confined population of India, is becoming one of the popular systems of well-being worldwide and one of the soft powers in nations' foreign policy. Such incredible turnaround has also tendered a responsibility to produce quality products along with evidence-based therapeutics convincing the swift modification in the approach towards research and researchers community [1].

Quality research is not possible until a researcher strives to expand a research culture and the institutional environment for research is established. A research ecosystem allows the research community to communicate, exchange, and transmit knowledge, which can then autonomously mentor future generations of researchers. Research aptitude is one of the important traits that one possesses that can meet the criteria of being a researcher. This aptitude is to be developed by a learner during his professional education [2].

Research methodology as the subject was an integral part of post-graduation (PG) but in recent times, its elementary component is been integrated into the final professional of undergraduate (UG) curriculum of the medical education. A foretaste of research methodology just before graduation might not develop research aptitude in scholars as most of them just take it as an ephemeral subject rather than as developmental proficiency. In addition, there is a lack of procedural learning, rational discussion and research apprehensions among students in present-day institutional training.

Institutional journal clubs (JCs) can be the contemporary era wadding in students' lives to understand and develop the missing research aptitude. JCs can be described as a group of students, scholars, researchers or clinicians interacting periodically and rationally discussing a published article of common interest from reputed journals [3]. The JC run by Osler as per Cushing's report is quoted as the foremost testified journal club in modern medical education [4]. Such clubs slowly become part of medical education, especially in PG and research institutes all over the world.

JCs are not a novel entity in the field of higher education but their implementation in Ayurveda educational institutions is for sure uncustomary. The impending demand for evidence-based therapeutics and preventive aspects of Ayurveda can only be fulfilled by early introduction and developing research aptitude of undergraduate students. Familiarity with the research idea, protocol, type, design, recent findings/updates and its significance in the evolution of Ayurveda can be a game changer in the development of research aptitude. This could be achieved by a sincere interactive and cost-effective approach of JCs [5].

Implementation of JCs for UGs will be certainly different from the proceedings of PG clubs but the basic theme should be similar to standard proceedings so that the students when in future pursue PG should not feel alienated from the JCs format. The UG student does not belong to any speciality stream and has to undertake both theoretical and practical understanding of all the specified subjects as per curriculum; there have to be modifications in proceedings as well as outcomes of such activity. The sole purpose of conducting the journal article reading is to understand the idea of research, procedural learning, and the importance of scientific writing. The progression of implementation and activities of the JCs for UG is summarised in three phases as in Table 1.

**Table 1:** Proposed agendas and their activities for implementation of Journal clubs for UG Students

I. Preparatory phase				
SNO	Program/Agenda	Activity		
a.	Faculty Engagement and Logistics support	• Engaging and providing passionate faculty members to familiarize them with the journal club layout and its prerequisites. (Pre-recorded sessions YouTube channel of J-AIM Journal Club could be a great learning experience. https://www.youtube.com/@ccihayushcoe) • Ensure access to relevant journals or databases. • The place of the meeting can be Seminar Halls/Auditoriums/Garden etc (If possible avoid regular lecture theatres/rooms) • Presentation equipment (Projector/Computer etc)		
b.	Meeting Schedule	• Once in a month. • Schedule timing for non-lecture classes (NLC) can be utilized		
II. Journal Club Sessions				
c.	Orientation Session	• An orientation session is to be arranged to explain the purpose and structure of the journal club. Clearly outline the goals of the journal club like enhancing critical appraisal skills, fostering research aptitude, etc • A social media group can be created for information purposes.		
d.	Article Selection	• Selecting articles that are under institutional subscriptions or open-access resources • Selecting relevant, impactful, and evidence-based research that aligns with Ayurvedic concepts, therapies, medicinal plants or latest advancements • Having methodological soundness (randomized controlled trials, systematic reviews or meta-analyses) • Having discussion potential can be prioritized.		
		Monthly Session		Bi-monthly Session
		1st Prof.	Reviews, latest advancements, Standardization of classical terms/values, latest updates in Rachana, Kriya or other concern topics conducted jointly by students and faculty (procedural learning)	• Rotating through various topics within Ayurveda—herbal medicine, Panchakarma, Interdisciplinary Approach, dietetics, etc., to provide a comprehensive view. (Focus on studies demonstrating positive patient outcomes or evidence supporting traditional Ayurvedic treatments) • Will be conducted by Final professionals and interns. (Students of 1st & 2nd Professionals will be in the audience and may ask questions if they have any queries.)
		2nd Prof.	Standardization of classical terms, latest advancements, pharmacological studies, investigational/lab studies, latest updates of Dravya Guna, Roga Nidana, Swasthvrita or other concern topics conducted jointly by students and faculty (procedural learning & capacity development)	
		3rd Prof.	Case Studies, drug studies, Standardization of protocol, latest finding of Kayachikitsa, Panchkarma, Prasuti or other concern topics	
e.	Presentation, Discussion, Takeaway message	• Encourage active participation and rotation of responsibility for presenting articles among students under the mentorship of faculty. • Moderation should ensure that discussions remain focused and inclusive, fostering a culture where different opinions and interpretations are respected and debated constructively. • After deliberations, the faculty mentor or in-charge should delib erate the concluding remarks.		
III. Feedback & Sustainability				
f.	Evaluation and Feedback	• Regularly gathering of feedback after each session from participants to assess the effectiveness of sessions to refine the structure, content, and facilitation of the journal club.		
g.	Recognition and Rewards	• Appreciation and acknowledgement for active participation and quality contributions from students and faculty should be given by the college/institute.		
h.	Sustainability	• Ensure institutional support and commitment to sustaining the journal club long-term.		

Globally several efforts are been made to implement the JCs at the UG level at contemporary medical schools. Primarily considered to be the domain of PGs, the introduction and integration of JCs among UG scholars for early faceoff with research and its application; has been incessantly promoted. Many studies have shown positive results of journal clubs in developing research aptitude enhancing professional skills and updating recent scientific findings. High levels of satisfaction among students, both at learning and future researcher prospects, were found in a study conducted at the Faculty of Medicine, Al-Baha University to determine the effectiveness of JCs presentation as a learning modality in an integrative undergraduate medical curriculum adopted not only in the basic and preclinical years but also in the clinical year [6]. In a different cross-sectional survey of undergraduate medical students at a government medical college in Puducherry, India, to determine the prevalence and usage patterns of journals among students, it was found that about 80% of the participants thought journals should be part of undergraduate training programs [7]. Further, a cross-sectional study of undergraduate medical students at The National University of Science and Technology's College of Medicine and Health Sciences found that starting a journal club would improve students' capacity to learn continuously throughout their lives [8].

An informal journal article reading was conceded for the first professional BAMS scholars of the institute by circulating the articles on the concern ongoing topics of the class on social media groups. Presentations and deliberations were done during non-lecture class schedules; most of the students liked the idea as they had gone through the articles on a prior basis and cleared their doubts during deliberations. The best part was that several students themselves explored the articles that converse the topics in some other outlook or approach.

The expected outcome of successful and sustained implementation of journal clubs for UG students will be procedural learning, an improvement of rational thinking, an experience of enhanced collaborative learning, staying updated with Ayurvedic research, communication skills and in general enrichment of research aptitude [9,10]. On the other hand, the faculty involved in the execution of journal clubs will also have mentoring opportunities, continued learning, integrating research in their practice, updated with current research, and enhancing their teaching methodologies and Innovation. An institute offering such a platform will certainly develop the research culture imbibing research aptitude among students as well as faculty and in a true sense can help in developing evidence-based practice in Ayurveda.

## Funding sources

None.

## Author contribution

The author has single handedly contributed in writing and conceptualising the manuscript.

## Declaration of generative AI in scientific writing

None.

## Conflict of interest

The authors declare that they have no known competing financial interests or personal relationships that could have appeared to influence the work reported in this paper.
